# Use of Melatonin Is Associated With Lower Risk of Colorectal Cancer in Older Adults

**DOI:** 10.14309/ctg.0000000000000396

**Published:** 2021-08-03

**Authors:** Naiqi Zhang, Jan Sundquist, Kristina Sundquist, Jianguang Ji

**Affiliations:** 1 Center for Primary Health Care Research, Department of Clinical Sciences Malmö, Lund University, Sweden;; 2 Department of Family Medicine and Community Health, Department of Population Health Science and Policy, Icahn School of Medicine at Mount Sinai, New York, New York, USA;; 3 Center for Community-Based Healthcare Research and Education (CoHRE), Department of Functional Pathology, School of Medicine, Shimane University, Japan.

## Abstract

Preclinical evidence suggests that melatonin may affect cellular pathways involved in colorectal cancer (CRC). We sought to test whether melatonin use was associated with decreased risk of CRC using population-based data.

**METHODS:**

We performed a nationwide cohort study using a new-user study design. We identified a total of 58,657 incident melatonin users aged 50 years and older from the Prescribed Drug Register, and matched them with 175,971 comparisons who did not use melatonin, on the ratio of 1:3. The Cox regression model was used to calculate hazard ratios and 95% confidence intervals.

**RESULTS:**

The incidence rate of CRC was 10.40 per 10,000 person-years for melatonin users, whereas the rate was 12.82 per 10,000 person-years in the nonusers. We found a significant negative association between melatonin use and risk of CRC (adjusted hazard ratio, 0.82; 95% confidence interval, 0.72–0.92). A test for trend showed a significant dose-response correlation (*P* < 0.001). The decrease of CRC risk was independent of tumor location and stage at diagnosis. When stratified by age groups, the inverse association was significant only among individuals aged 60 years and older.

**DISCUSSION:**

This population-based cohort study suggests that the use of melatonin was associated with a reduced risk of CRC. Further studies are needed to confirm the observed association and to explore the underlying mechanisms.

## INTRODUCTION

Globally, colorectal cancer (CRC) remains the third most diagnosed cancer and the second leading cause of death through cancer. According to statistics from the International Agency for Research on Cancer, there were approximately 1.8 million new CRC cases and 900,000 deaths in 2018, accounting for about one-tenth of cancer cases and deaths (1). The incidence and mortality of CRC increase rapidly after age 50 years, leading to an increasing global burden in the foreseeable future because of population aging (2). Considering the relatively high risk of developing CRC in older adults, effective preventive strategies are highly needed among older adults. Chemoprevention, using medications to block the pathogenetic pathways of disease, has become an attractive strategy for cancer prevention. Over recent decades, several non-anti-cancer medications were found to have potential benefits regarding CRC prevention (3). Low-dose aspirin was recommended by the US Preventive Services Task Force for the primary prevention of CRC. However, the positive net benefit was obvious only in adults aged 50–59 years, whereas the harms (gastrointestinal bleeding as an example) may exceed the benefits for the older adults (4,5). Therefore, effective and safe chemoprevention is critical for reducing the incidence rate of CRC in older adults.

Melatonin is a natural indolic compound mainly secreted by the pineal gland of humans and mammals, which regulates the circadian rhythm (6). Clinically, melatonin is used orally for the short-term treatment of insomnia, such as jet lag or during shift work (7). Because circadian rhythm disruption was found to be a contributing factor in cancer development (8), melatonin has attracted great attention in cancer prevention and adjuvant cancer treatment. Besides the important role in regulating the circadian rhythms, melatonin is also acknowledged for its antioxidant, anti-inflammatory, immune-modulating, and oncostatic activities (9–11). Several epidemiological studies support a protective role of melatonin in breast (12–16), prostate (17,18), and ovarian cancers (19), yet other studies yielded controversial conclusions (20–23). However, population-based evidence for the association of melatonin with CRC remains lacking. Furthermore, previous epidemiologic studies mainly concerned body melatonin levels, such as urinary melatonin exertion or serum melatonin levels; none focused on its chemopreventive effect as oral medication.

Therefore, the objective of this study was to investigate whether the use of melatonin was associated with a reduced CRC incidence in the elder Swedish population (age 50+ years). We hypothesized that melatonin might have a protective effect regarding the development of CRC.

## METHODS

### Study population

This retrospective nationwide cohort study was approved on February 6, 2013, by the Ethics Committee at Lund University (Dnr 2012/795), Sweden. We used information on individuals from Swedish population-based registers with national coverage. These registers were linked using each person's unique identification number replaced by a serial number to preserve confidentiality.

**Figure 1.**
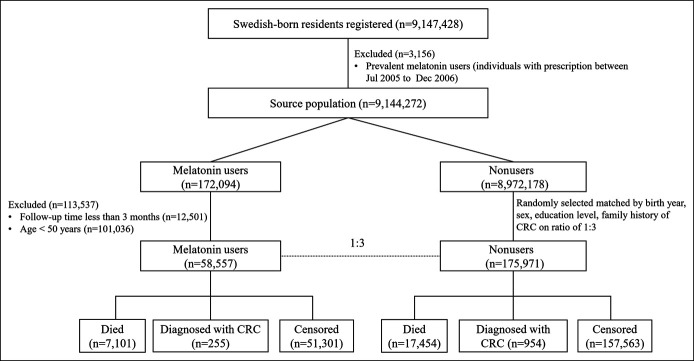
Flow chart of participants involved in this national cohort study. CRC, colorectal cancer.

The source population included 9,147,428 cancer-free Swedish-born residents registered from July 2005 through December 2015 in the Swedish Total Population Register, which includes detailed demographic information for nearly 100% of residents living in Sweden (24). The Swedish Prescribed Drug Register was created on July 1, 2005, and includes data on all prescribed drugs dispensed at pharmacies covering the entire Swedish population (25). The rate of missing individual identity data is estimated to be lower than 0.3% (26). We used the Swedish Prescribed Drug Register to identify individuals, who were ever prescribed melatonin from July 2005 through December 2015, according to Anatomical Therapeutic Chemical Classification code N05CH. We excluded individuals younger than 50 years at first prescription and individuals whose follow-up times were less than 3 months (Figure 1). To adopt a new-user study design, the cohort entry date was set as January 1, 2007; thus, a washout period of 1.5 years was applied. Individuals, who previously had any prescription of melatonin before the entry date (n = 3,156), were not included in this study.

For each melatonin user, 3 individuals without a prescription of melatonin were randomly selected from the source population and matched by birth year, sex, education level, and family history of CRC.

### Outcome measurement

We further linked these individuals to the Swedish Cancer Registry (27) to identify patients who had been diagnosed with CRC from July 1, 2007, through December 31, 2016, by using the *International Classification of Diseases, Tenth Revision, Clinical Modification* codes C18, C19, and C20. The Swedish Cancer Registry contains data on the TNM staging system, including the size of the tumor (T), nodal status (N), and presence of metastatic disease (M). By combining the T, N, and M categories, we can determine the stage at diagnosis of CRC, ranging from stage I (the least advanced) to stage IV (the most advanced) as follows: stage I (T1 or T2, N0, and M0), stage II (T3 or T4, N0, and M0), stage III (any T, N1 or N2, and M0), and stage IV (any M1) (28). By further linking to the Cause of Death Register, we could identify individuals who had died during the follow-up period.

The follow-up was started on the date of the first prescription of melatonin for melatonin users or the index date for their comparisons (the same date as the corresponding melatonin user), ended at the first date of diagnosis of cancer, date of death from any cause, and the end of the study period (December 31, 2016), whichever came first.

### Assessment of covariates

By retrieving data from the National Patient Register, Statistics Sweden's Total Population Register and Population Housing Census, and Swedish Multiple Generation Register, we extracted information on potential confounding factors, including birth year, sex (male or female), highest education (1–9, 10–11, and ≥12 years) (25), family history of CRC (having at least 1 first-degree relative diagnosed with CRC, yes/no), history of inflammatory bowel disease (Crohn's disease or ulcerative colitis, yes/no), history of colonoscopy (yes/no), obesity (identified from the National Patient Register using *International Classification of Diseases-10* code E66, yes/no), chronic obstructive pulmonary disease (COPD, yes/no) as a proxy for smoking, prescription of other medication (aspirin, statin, and metformin, yes/no), and Charlson Comorbidity Index (CCI; 0, 1, 2, and ≥3). As having comorbidity is an important factor affecting the health condition and risk of cancer, we calculated the CCI based on a total of 17 categories.

### Statistical analysis

We used a competing risk Cox regression model to calculate hazard ratios (HRs) and 95% confidence intervals (CIs) of CRC associated with melatonin use to control the competing risk of death (29). The χ^2^ test was used to test the difference of sociodemographic and clinical factors between individuals who used melatonin and the matched comparisons, and variables with *P* values < 0.1 were included in the regression model. The final model was adjusted for the history of inflammatory bowel disease, use of colonoscopy, outpatient visits, obesity, COPD, use of aspirin, use of statin, and use of metformin besides the matched factors. Moreover, we evaluated the association of melatonin use with the risk of the specific site of cancer (proximal colon, distal colon, and rectum) and specific stage of cancer (stage I or II and stage III or IV). In addition, we calculated the cumulative defined daily doses (cDDDs) of melatonin as the sum of DDDs for all prescriptions during the follow-up period using records from the Swedish Prescribed Drug Register. We then performed a dose-response analysis of melatonin use with CRC by modeling cDDD of melatonin as 3 groups: <30 cDDD, 30–89 cDDD, and ≥ 90 cDDD and tested for the trend by entering the cDDD as a continuous variable in the regression model. We further conducted several sensitivity analyses to explore the possibility of chance findings. First, we performed an analysis by lagging exposure to melatonin for 1 year to evaluate whether the results of the main analysis could be influenced by biological latency. Second, to minimize reverse causation, we excluded CRC diagnosed within 4 years after the baseline date. Third, we evaluated the risk of CRC among individuals aged 50 years and older, after excluding individuals with a history of benign colorectal tumor. Fourth, to increase confidence in the reported association, we evaluated a negative control outcome (incident diabetes). In addition, we performed a sensitivity analysis to evaluate the influence of potential misclassification of melatonin use, and the sensitivity and specificity were defined as 88% and 99%, respectively.

All analyses were conducted using SAS, version 9.4 (SAS Institute, Cary, NC).

## RESULTS

From the Swedish Prescribed Drug Register, a total of 172,094 incident melatonin users were identified between July 1, 2007, and December 31, 2015 (Figure 1). Among them, we further identified 58,657 eligible individuals aged 50 years and older, and matched them with 175,971 comparisons, who did not use melatonin on the ratio of 1:3. The distribution of matched factors was similar in melatonin users and comparisons (*P* > 0.05). Compared with individuals without melatonin use, incident melatonin users had a higher proportion of inflammatory bowel disease, obesity, COPD, colonoscopy screening, aspirin, metformin and statin use, and higher CCIs (Table 1). These variables were adjusted in the final multivariable regression model.

**Table 1.** Demographic and clinical characteristics among melatonin users and matched comparisons aged 50 years and older at the index date

	Melatonin users (N = 58,657)		Nonusers (N = 175,971)		*P* value
	No.	%	No.	%	
Age at index					1.00
50–59 yr	19,139	32.6	57,417	32.6	
60–69 yr	17,551	29.9	52,653	29.9	
≥70 yr	21,967	37.4	65,901	37.4	
Sex					1.00
Male	19,621	33.5	58,863	33.5	
Female	39,036	66.5	117,108	66.5	
Highest education level, yr					1.00
1–9	13,337	22.7	40,011	22.7	
10–11	22,224	37.9	66,672	37.9	
≥12	23,096	39.4	69,288	39.4	
Family history of CRC					1.00
No	54,122	92.3	162,366	92.3	
Yes	4,535	7.7	13,605	7.7	
Inflammatory bowel disease					<0.001
No	57,690	98.4	173,864	98.8	
Yes	967	1.6	2,107	1.2	
Obesity					<0.001
No	57,032	97.2	173,249	98.5	
Yes	1,625	2.8	2,722	1.5	
COPD					<0.001
No	52,462	89.4	163,426	92.9	
Yes	6,195	10.6	12,545	7.1	
Colonoscopy					<0.001
No	54,787	93.4	168,785	95.9	
Yes	3,870	6.6	7,186	4.1	
CCI					<0.001
0	37,516	64.0	126,987	72.2	
1	13,035	22.2	30,480	17.3	
2	4,642	7.9	11,038	6.3	
≥3	3,464	5.9	7,466	4.2	
Prescription of other medicines					
Aspirin	14,321	24.4	35,789	20.3	<0.001
Metformin	3,661	6.2	10,011	5.7	<0.001
Statin	16,462	28.1	41,618	23.7	<0.001

CCI, Charlson Comorbidity Index; COPD, chronic obstructive pulmonary disease; CRC, colorectal cancer.

After an accumulated 989,445 years of follow-up, the incidence rate of CRC among older people who had ever used melatonin was 10.40 per 10,000 person-years, which was lower than comparisons who did not use melatonin (incidence rate, 12.82 per 10,000 person-years). Melatonin use was inversely associated with CRC risk among older adults, with a crude HR of 0.80 (95% CI, 0.71–0.91) and an adjusted HR of 0.82 (95% CI, 0.72–0.92). We found the inverse association was slightly stronger for rectal cancer (adjusted HR, 0.73; 95% CI, 0.58–0.93) compared with distal colon cancer (adjusted HR, 0.89; 95% CI, 0.74–1.08) and proximal colon cancer (adjusted HR, 0.80; 95% CI, 0.63–1.01) and for earlier stages (adjusted HR, 0.78; 95% CI, 0.65–0.94) compared with advanced stages (adjusted HR, 0.82; 95% CI, 0.68–0.99) (Table 2).

**Table 2.** HRs and 95% CIs of CRC risk associated with melatonin use

	Individuals, n	Person-years	CRC diagnoses, n	IR, per 10,000 person-years	Crude			Adjusted^a^		
					HR	95% CI	*P* value	HR	95% CI	*P* value
Ever use of melatonin										
No	175,971	744,141	954	12.82	1			1		
Yes	58,657	245,304	255	10.40	0.80	0.71–0.91	<0.001	0.82	0.72–0.92	0.001
Cancer site										
Proximal colon										
Nonusers	175,971	744,141	380	5.11	1			1		
Melatonin users	58,657	245,304	112	4.57	0.88	0.74–1.06	0.184	0.89	0.74–1.08	0.237
Distal colon										
Nonusers	175,971	744,141	272	3.66	1			1		
Melatonin users	58,657	245,304	71	2.89	0.78	0.62–0.99	0.037	0.80	0.63–1.01	0.058
Rectum										
Nonusers	175,971	744,141	283	3.80	1			1		
Melatonin users	58,657	245,304	66	2.69	0.70	0.55–0.89	0.004	0.73	0.58–0.93	0.011
Stage at cancer diagnosis										
Stage I or II										
Nonusers	175,971	744,141	418	5.62	1			1		
Melatonin users	58,657	245,304	110	4.48	0.79	0.66–0.95	0.013	0.78	0.65–0.94	0.010
Stage III or IV										
Nonusers	175,971	744,141	420	5.64	1			1		
Melatonin users	58,657	245,304	110	4.48	0.78	0.65–0.94	0.010	0.82	0.68–0.99	0.041

CCI, Charlson Comorbidity Index; CI, confidence interval; COPD*,* chronic obstructive pulmonary disease, CRC, colorectal cancer; HR, hazard ratio; IR, incidence rate.

a Adjusted for age at index, sex, education, family history of CRC, personal history of inflammatory bowel disease, use of colonoscopy, obesity, COPD, CCI, use of aspirin, use of statin, and use of metformin.

**Figure 2.**
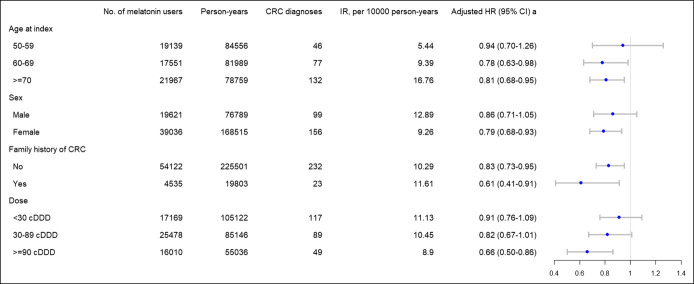
Hazard ratios and 95% CIs of CRC risk associated with melatonin use stratified by age at index, sex, family history of CRC, and doses of melatonin use. ^a^Adjusted for age at index, sex, education, family history of CRC, personal history of inflammatory bowel disease, use of colonoscopy, obesity, COPD, CCI, use of aspirin, use of statin, and use of metformin. CCI, Charlson Comorbidity Index; CI, confidence interval; COPD*,* chronic obstructive pulmonary disease, CRC, colorectal cancer; HR, hazard ratio; IR, incidence rate.

The results of stratified analyses are listed in Figure 2. We observed a significant inverse association between melatonin use and CRC risk among individuals aged 60 years and older (adjusted HR and 95% CI, 0.78 and 0.63–0.98 for the age group 60–69 years; 0.81 and 0.68–0.95 for the age group ≥ 70 years), but not significant in the age group 50–59 years (adjusted HR, 0.94; 95% CI, 0.70–1.26). When stratified by sex, melatonin use was associated with a lower CRC risk among women (adjusted HR, 0.79; 95% CI, 0.68–0.93), but not significant among men (adjusted HR, 0.86; 95% CI, 0.71–1.05). The risk of CRC was 0.91 (95% CI, 0.76–1.09) among patients with a cumulative dose of melatonin less than 30 cDDD, and it decreased to 0.82 (95% CI, 0.67–1.01) among patients with a cumulative dose of 30–89 cDDD and 0.66 (95% CI, 0.50–0.86) among patients with the highest cumulative dose 90 cDDD and above. Test for the trend of the dose-response correlation showed significant results (*P* < 0.001).

Sensitivity analyses' results are listed in Table 3. In sensitivity analysis 1, the use of melatonin continued to be associated with a reduced risk of CRC (adjusted HR, 0.71; 95% CI, 0.67–0.74) after being lagged for 1 year after the first administration of melatonin. In sensitivity analysis 2, we excluded CRC patients diagnosed within 4 years after the baseline date; the results stayed robust with the main analysis (adjusted HR, 0.70; 95% CI, 0.56–0.88). In sensitivity analysis 3, melatonin use continued to be associated with a decreased risk of CRC among older adults after excluding patients with a history of benign colorectal tumor, with an adjusted HR of 0.83 (95% CI, 0.73–0.94). In sensitivity analysis 4, melatonin use was not associated with incident diabetes (adjusted HR, 1.04; 95% CI, 0.97–1.11). In sensitivity analysis 5, after correcting for misclassification of melatonin use, the estimated corrected HR was 0.79, with 95% CI of 0.68–0.91.

**Table 3.** Summary of the sensitivity analyses

	Individuals, n	Person-years	Disease diagnoses, n	IR, per 10,000 person-years	Crude			Adjusted^a^		
					HR	95% CI	*P* Value	HR	95% CI	*P* Value
Sensitivity analysis 1^b^										
Nonusers	154,610	730,644	766	10.48	1			1		
Melatonin users	51,004	240,513	205	8.52	0.79	0.68–0.89	<0.001	0.79	0.69–0.91	<0.001
Sensitivity analysis 2^c^										
Nonusers	85,905	573,334	362	6.31	1			1		
Melatonin users	28,635	190,972	77	4.03	0.64	0.51–0.80	<0.001	0.70	0.56–0.88	0.002
Sensitivity analysis 3^d^										
Nonusers	172,731	735,730	917	12.46	1			1		
Melatonin users	57,577	242,469	249	10.27	0.82	0.72–0.92	0.001	0.83	0.73–0.94	0.003
Sensitivity analysis 4^e^										
Nonusers	139,905	554,484	3,044	54.90	1			1		
Melatonin users	46,635	182,972	1,111	60.72	1.09	1.01–1.17	0.016	1.04	0.97–1.11	0.321
Sensitivity analysis 5^f^										
Corrected nonusers	64,725	744,141	954	12.82	—			1		
Corrected melatonin users	169,903	245,304	255	10.39				0.79	0.68–0.91	<0.001

CCI, Charlson Comorbidity Index; CI, confidence interval; COPD*,* chronic obstructive pulmonary disease, CRC, colorectal cancer; HR, hazard ratio; IR, incidence rate.

a Adjusted for age at index, sex, education, family history of CRC, personal history of inflammatory bowel disease, use of colonoscopy, obesity, COPD, CCI, use of aspirin, use of statin, and use of metformin.

b Sensitivity analysis 1: risk of CRC among individuals aged 50 years and older. The time to follow-up was defined as 1 year after the first use of melatonin.

c Sensitivity analysis 2: risk of CRC among individuals aged 50 years and older after excluding CRC diagnosed within 4 years after the baseline date.

d Sensitivity analysis 3: risk of CRC among individuals aged 50 years and older after excluding individuals with benign colorectal tumor.

e Sensitivity analysis 4: risk of diabetes among individuals aged 50 years and older.

f Sensitivity analysis 5: results of probabilistic sensitivity analyses correcting for misclassification of melatonin use (sensitivity = 0.88 and specificity = 0.99).

## DISCUSSION

As far as we know, this retrospective nationwide cohort study is the first population-based study to explore whether the use of melatonin was associated with a reduced CRC risk in older adults. We found a statistically significant inverse association between melatonin use and CRC incidence among older adults. In addition, the reduced risk was associated with an increased cumulative dose of melatonin use, and individuals with the highest doses have a 34% reduced risk of developing CRC. The association was apparent irrespective of CRC location and cancer stage at diagnosis. Our study highlights the possibility of melatonin as a potential chemopreventive agent for the primary prevention of CRC from nationwide real-world data.

Melatonin is widely known for the functionality of regulating night and day cycles or sleep-wake cycles. Besides the pineal gland, the digestive tract is also a rich source of melatonin. Gut melatonin is mainly synthesized in serotonin-rich enterochromaffin cells of the mucosa and submucosa of the human jejunum and colon (30). Although the production of melatonin in the pineal gland is inhibited by exposure to light and activated during darkness, the release of melatonin in the gastrointestinal tract seems to be dependent on food consumption and specific gut bacteria (31,32). Instead of releasing into the blood as opposed to melatonin produced in the pineal gland, gut melatonin acts locally as an autocrine and/or paracrine signal (33). The concentration of melatonin in the gastrointestinal tissues surpasses blood levels by 10- to 100-folds and surpasses pineal gland level by 400 times (34). This large quantity of gastrointestinal melatonin plays a role in active regulation of regeneration and function of gastrointestinal epithelium and enhancing the immune activity of gut (32,35). Although the biological mechanisms of melatonin against CRC are not completely established, some hypotheses have been put forward and validated *in vivo* or *in vitro* models. First, melatonin presented an oncostatic effect in colon cancer cells, which might partly be because of its antioxidative and anti-inflammatory activities (36). Melatonin could reduce the expression of nitric oxide synthase and increase the expression of glutathione peroxidase and reductase, thus downregulating the oxidative stress and decreasing mutagenesis and cell proliferation in colorectal carcinoma cell lines (37,38). Second, melatonin was reported to enhance apoptosis of CRC cells possibly through inducing dephosphorylation and nuclear import of histone deacetylase 4 and subsequent H3 deacetylation through the inactivation of Ca2+/calmodulin-dependent protein kinase IIα (39). Third, evidence from experiments *in vitro* suggested that melatonin exerted an antitumor effect through activating cell death programs and cellular senescence (40), inhibiting cell proliferation (41), and inducing morphological changes (42) in colorectal carcinoma cell lines. Fourth, melatonin ameliorated the progression of colon carcinogenesis by inhibiting autophagy and increasing the expression of Nrf2 and the associated antioxidant enzymes, thus consequently decreased inflammation, oxidative stress, and DNA damage in the colon in a mouse model (43). Fifth, the anti‐CRC action of melatonin seems to depend on the status of specific membrane receptors, melatonin receptor type-1 (MT1) and melatonin receptor type‐2 (MT2). Several studies reported downregulation of MT1 and MT2 translation and transcription in colorectal tumors compared with healthy adjacent mucosa, indicating that the invasion capacity of colorectal tumor cells is correlated with the expression of MT1 and MT2 (44–46). Furthermore, melatonin is likely to affect cancer stem cells in CRC. The combination of 5-FU and melatonin stimulated apoptosis and autophagy in colon cancer stem cells by regulating cellular prion protein-Oct4 axis (47,48).

Unfortunately, we could not disentangle whether the observed association was due to the antitumor effect of melatonin or the benefit from good quality sleep. Available evidence suggests that a higher level of melatonin in the blood associates with sleep propensity during the night, but the association seems not being causal (49). Results from healthy human subjects and rat models suggested that sleep deprivation and exposure to a constant light environment did not suppress melatonin serum levels despite their associations with the disruption of the melatonin rhythms (50,51). However, melatonin supplementation could reverse the preneoplastic changes and restore the blockade on melatonin receptors induced by a constant light environment. Melatonin supplementation could also control the proliferation of preneoplastic cells and induce a higher expression of caspase-3 protein (52).

To the best of the authors' knowledge, no epidemiological study has yet been published to evaluate the association between melatonin administration and CRC risk. A population‐based case‐control study reported prolonged night‐shift work among men could increase the risk of CRC, indicating that a regular circadian melatonin pattern would seem to be oncopreventive for humans under chronic light pollution (53). This retrospective cohort study, based on data from high-quality complete nationwide registers in Sweden, provides the first population-based evidence of the anticancer effect of melatonin on CRC among older adults. Epidemiological studies concerning the association between body circadian melatonin levels and cancer risk drew controversial conclusions. Two meta-analyses investigated the association between urinary excretion of melatonin and breast cancer suggested that the increase in urinary melatonin excretion was associated with a reduced breast cancer risk, with a linear dose-response trend (13,14). Two case-control studies reported a significant inverse correlation between urinary melatonin level and risk of prostate cancer in men (17,18). A retrospective study focused on ovary cancer found that the serum levels of melatonin were significantly lower in patients with ovarian cancer compared with healthy controls (19). However, another study reported no association between urinary melatonin level and ovarian cancer (23). Results from a series of clinical trials showed that melatonin can be an efficient and cost-effective adjuvant of cancer treatment. With a concomitant daily administration of melatonin, the efficacy of cytotoxic drugs in the chemotherapy was enhanced in metastatic CRC patients (54). Besides CRC, the concurrent use of melatonin in the treatment of various solid cancers could enhance the therapeutic efficacy, reduce the toxicities of other anticancer drugs to improve tolerance to chemotherapy, relieve the side effect of chemotherapies or radiation, and significantly improve the 1- and 5-year survival rate (55,56). Based on the evidence mentioned above, the impressive efficacy of melatonin supports it as a promising chemoprevention agent against CRC.

There might be a concern about the safety of melatonin as a chemoprevention agent. Because melatonin is an endogenously produced hormone, it is well tolerated at physiological concentrations. It is widely used in humans as a sleep aid, and no acute or chronic toxicities were reported. Evidence from a systematic review showed that most of the side effects were minor and short (fatigue, headache, and sleepiness), whereas more serious side effects (increasing in systolic and diastolic blood pressure and heart rate) were seen at very high doses or in specific populations. Most untoward effects can be easily managed by dosing in accordance with natural circadian rhythms (57). Future well-designed randomized clinical trials are highly needed to confirm the antitumor effect, and the safety profile before melatonin can be used as a chemopreventive agent.

The major strength of the current study was that it was performed based on the population at a national level. The cohort study design and large sample size ensured statistical power and avoided reversal causality. Data collected by continuously updated nationwide registers could efficiently eliminate recall bias and minimize selection bias. Register-based data also provided us with information on potential demographic and clinical confounding factors. We selected comparisons matched by birth year, sex, education level, and family history of CRC, while other confounders were included in the regression models. The design also allowed the assessment of a dose-response effect for melatonin exposure on CRC. A few limitations warrant consideration. First, the results might be affected by detection bias because individuals who used melatonin may be more likely to have healthier lifestyles; thus, they were more likely to screen for CRC and might be diagnosed with CRC at an earlier stage. However, our regression models adjusted colonoscopy, which is offered in Sweden in an opportunistic manner and can be seen as an indication of health behavior because people with higher levels of knowledge and higher health awareness are more likely to have colonoscopy (58). Therefore, the adjustment of colonoscopies might partly exclude the contribution of different health behavior among individuals use melatonin and those without using it. In addition, our stratification analyses showed that the association was independent of the clinical stage at diagnosis, suggesting such detection bias might play a small role in our observation. Second, we lack information on some potential confounding factors, such as smoking, alcohol drinking, and dietary factors in our nationwide databases. However, we have adjusted for COPD in regression models. Although it is a crude proxy for smoking, it might partly exclude the confounding effect of smoking. We additionally adjusted for education status, which was highly associated with lifestyle factors and might partly exclude their confounding effect. Third, the results might be influenced by misclassification bias because melatonin is available over the counter in Sweden. However, the price for melatonin over the counter is much higher than that prescribed by clinicians in Sweden; thus, melatonin users are inclined to have their medication prescribed, which we can identify from the register. In addition, we conducted a sensitivity analysis to correct for misclassification of melatonin exposure, and the result was consistent with that of the main analysis. Fourth, because of the relatively short follow-up time in this study, exploring the anticancer effect among young adults was precluded because of the small number of cases. Thus, future studies with longer follow-up and larger sample size could be considered to explore this subject further.

In conclusion, this population-based retrospective cohort study suggests that the use of melatonin was associated with a reduced risk of CRC, and the decrease showed a dose-response correlation. Findings from this study need to be confirmed by well-designed randomized clinical trials in the future.

## CONFLICTS OF INTEREST

**Guarantor of the article:** Jianguang Ji, MD, PhD

**Specific author contributions:** N.Z., J.S., K.S., and J.J.: study concept and design. J.S., K.S., and J.J.: obtained funding. K.S. and J.S.: data acquisition. N.Z.: statistical analysis and drafting the manuscript. All authors revised it for important intellectual content.

**Financial support:** This work was supported by grants awarded to J.J. by the Swedish Research Council (2016–02373), Cancerfonden (CAN2017/340) and Crafoordska Stiftelsen and Allmänna Sjukhusets i Malmö Stiftelsen för bekämpande av cancer, to K.S. by the Swedish Research Council (2018–02400), and to J.S. (2020–01,175) by the Swedish Research Council as well as by ALF funding from Region Skåne awarded to K.S., and J.J., and to N.Z. by China Scholarship Council (Grant No. 201906380063).

**Potential competing interests:** None to report.

**Study Highlights**

**WHAT IS KNOWN**

✓ Experimental studies suggested that melatonin had anticancer activity.
✓ Population-based evidence is poorly documented.

**WHAT IS NEW HERE**

✓ Melatonin use was associated with a reduced risk of colorectal cancer among older adults.
✓ The chemopreventive effect showed a dose-response pattern.
